# Survey of local impacts of biofuel crop production and adoption of ethanol stoves in southern Africa

**DOI:** 10.1038/sdata.2018.186

**Published:** 2018-09-18

**Authors:** Alexandros Gasparatos, Graham P. von Maltitz, Francis X. Johnson, Carla Romeu-Dalmau, Charles B. L. Jumbe, Caroline Ochieng, Shakespear Mudombi, Boubacar Siddighi Balde, Davies Luhanga, Paulo Lopes, Anne Nyambane, Marcin P. Jarzebski, Katherine J. Willis

**Affiliations:** 1 University of Tokyo, Tokyo, Japan; 2 Council for Scientific and Industrial Research (CSIR), Pretoria, South Africa; 3 Stockholm Environment Institute (SEI), Stockholm, Sweden; 4 University of Oxford, Oxford, UK; 5 Lilongwe University of Agriculture and Natural Resources, Lilongwe, Malawi; 6 Royal Botanical Gardens Kew, Richmond, UK

**Keywords:** Agriculture, Energy supply and demand, Environmental chemistry

## Abstract

The two datasets outlined in this paper contain information related to (a) the local impacts of biofuel feedstock production, and (b) the factors that influence the adoption and/or sustained use of ethanol stoves in southern Africa. The first dataset was generated through extensive household surveys around four operational jatropha and sugarcane production sites in Malawi, Mozambique, and Swaziland. This project aimed to examine the local impacts of the most prominent modes of existing or intended biofuel feedstock production in southern Africa. The resulting dataset contains information about impacts on rural livelihoods, ecosystem services, food security and poverty alleviation. The second dataset is the outcome of research into factors that influence the adoption and sustained use of ethanol stoves. This dataset was collected through a household survey in Maputo city where the only large-scale ethanol stove dissemination programme in Africa has been implemented.

## Background and Summary

Biofuels have been promoted in Africa predominately for energy security, rural development and other economic reasons^1^. While in some southern African countries the interest in biofuels for transport dates back to the late 1970s, only Malawi has played a pioneering role in the biofuel industry. In Malawi the blending of sugarcane ethanol with gasoline has occurred continuously since 1982 (ref. 2). However, the high oil prices of the mid-2000s catalyzed the significant revival of the interest for transport biofuels throughout Africa. While jatropha has been the most widely promoted biofuel feedstock across the continent since this time^1^, sugarcane production has also expanded substantially in some regions^3,4^. The underlying policy goals such as energy security and rural development have varied between countries^1^, and have dictated to a large extent how jatropha and sugarcane production has been approached, particularly in southern Africa. Large-scale plantations, smallholder-based projects, or hybrids of the two being the dominant modes of production^1,5,6^.

Given its land-intensive nature^5^, biofuel feedstock production can have profound environmental and socioeconomic impacts, especially in the poor agrarian contexts of Africa^1^. For example, different configurations of agricultural and natural areas are converted for biofuels feedstock production^5^. This land use and cover change (LUCC) can affect directly and indirectly the flow of various provisioning (e.g. food, timber, non-timber woodland products), regulating (e.g. carbon sequestration, water purification) and cultural (e.g. religious values) ecosystem services^5,7–9^. Changes in the flow of such ecosystem services can have substantial effects on human wellbeing and poverty alleviation, especially in the poor rural areas in Africa where local communities depend highly on ecosystem services for their livelihoods^9–11^.

Although there have been some studies on the local impacts of biofuel feedstock production in Africa, this literature tends to be fragmented. For example, some studies focus only on a single biofuel project, a limited set of impacts, a single mode of production, and/or a single type of feedstock. A few studies have assessed and compared the multiple impacts of large-scale and smallholder-based feedstock production systems in Africa^12,13^. Such studies, however, tend to use different research protocols for data collection and analysis, which hampers to a large extent the ability to compare between studies and develop a comparative understanding of the local impacts of feedstock production.

Biofuel/feedstock production in Africa has mainly targeted the transport sector, either domestically (e.g. sugarcane ethanol in Malawi) or for export into foreign markets (e.g. jatropha-based fuels in most other national contexts)^1^. Very few biofuel initiatives have produced fuel for non-transport purposes, such as rural electrification^14^ or cooking^15^. In addition, these initiatives had limited scope, mostly being pilot projects funded through foreign assistance to provide locally customized energy solutions. While, biofuels for cooking can offer a much cleaner and safer alternative to traditional charcoal and fuelwood stoves, the only such large-scale and market-led initiative in Africa is in Maputo, Mozambique^16^. However, to date there have been no studies to assess the successful take-up and implementation of ethanol stoves.

The above suggests the significant existing gaps related to the local impacts of biofuel production in southern Africa. Currently, there are no comprehensive comparative analyses of the different feedstock production configurations in the region. Furthermore, there is limited understanding of the impacts of operational jatropha projects due to the almost total collapse of the jatropha sector in southern Africa^17^. Most of the earlier jatropha studies focused on the initial project phases, and thus were either based on assumptions or were conducted before the true long-term impacts manifested. Furthermore, there are practically no studies on the factors that influence the adoption and sustained use of ethanol stoves in market settings, given that most of the current studies have focused on pilot stove dissemination projects.

This manuscript outlines two datasets that resulted from a research project aiming to address the existing knowledge gaps in (a) the local impacts of biofuel feedstock production, and (b) the factors that influence the adoption and sustained use of ethanol stoves. The first dataset was collected through extensive household surveys around four operational feedstock production sites in Malawi, Mozambique and Swaziland. These surveys targeted groups with different levels of involvement in feedstock production (e.g. plantation workers, smallholders), as well as groups not involved in feedstock production (control groups). The dataset contains information related to the impact of feedstock production on rural livelihoods, ecosystem services, food security, and poverty alleviation. To allow for some degree of comparability between sites, we followed as much as possible, the same research protocol at each location. Questionnaires were largely the same between sites for groups with similar level of involvement in feedstock production. However, the sampling protocol varied due to the specific project characteristics and information available. The second dataset includes information on the factors that influence the adoption and sustained use of ethanol stoves. This dataset was collected through a household survey in Maputo city, where the only large-scale ethanol stove dissemination programme in Africa has been implemented to date.

## Methods

### Study site selection

The first step towards the development of the datasets was the careful selection of study sites and participants. Initially, the research team conducted an extensive literature review^1,18^, which identified that: (a) jatropha and sugarcane have been the main biofuel feedstocks promoted in Africa, and (b) large-scale plantations, smallholder-based projects and hybrids between the two (mainly for sugarcane) have been the main modes of feedstock production. The literature review also revealed that the associated LUCC effects dictate largely the type and magnitude of impacts. The conversion of different land classes (e.g. forestland or agricultural land) can have quite different impacts^5^. Furthermore, the rules of smallholder engagement in feedstock value chains can also influence land allocation, received income, and payment structure, all of which are important factors that affect the actual human wellbeing outcomes of feedstock production^1^.

Following the review process, we selected four project sites as case studies:

- a hybrid sugarcane project that contained a large-scale sugarcane plantation surrounded by sugarcane smallholders (both rainfed and irrigated) in Dwangwa, Malawi;
- a site that contained a large-scale sugarcane plantation and several smaller community-owned plantations in Tshaneni, Swaziland;
- a smallholder-based jatropha project in Mangochi, Malawi;
- a large-scale jatropha plantation in Buzi, Mozambique.

Based on the literature review, the selected case studies represent the most prevalent biofuel feedstocks, modes of production and LUCC transitions in Africa. The selected case studies also represent diverse market engagement practices among smallholders, ranging from typical outgrower approaches (i.e. individual farmers sell feedstock directly to a sugarcane mill in Dwangwa, Malawi) to more cooperative models. In such cooperative models farmers have consolidated their lands into larger community plantations, becoming equal partners and receiving equity/dividends from sugarcane production (Tshaneni, Swaziland).

We focused on southern Africa as jatropha and sugarcane have both been promoted in the region, though with different national experiences. For example Malawi and Swaziland have a long history of sugarcane production^2,19^, while Mozambique only recently promoted jatropha for biofuels^20^.

**Figure 1:**
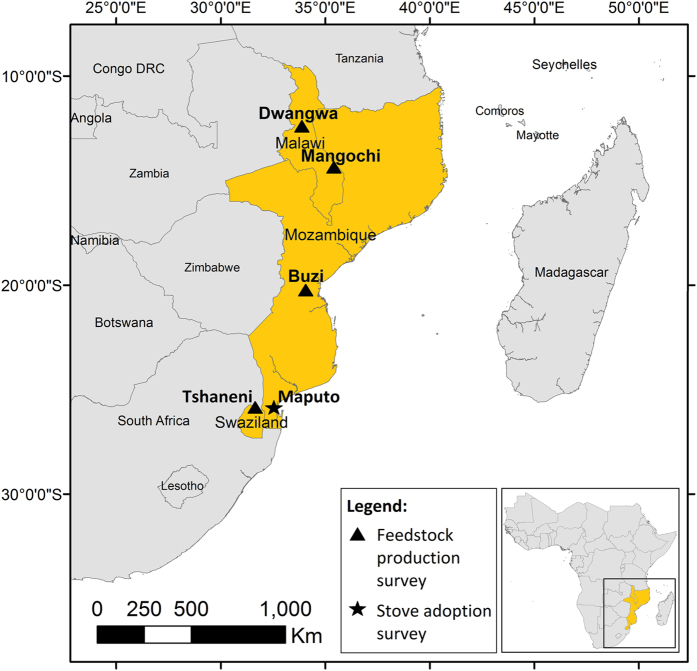
Location of study sites.

To allow for some level of impact manifestation, we also selected feedstock production projects that had been operational for at least five years prior to data collection. This complicated the selection of jatropha projects considering that the crop was extensively promoted only around 2005 (ref. 1); and that by the time of data collection in 2014–2015, most of the jatropha projects in southern Africa had already collapsed^17^. Both of the selected jatropha projects, however, met the inclusion criteria, as they had been continuously operational for at least 5 years before data collection-with regular payments made to the involved smallholders and plantation workers. Table 1 outlines some of the main characteristics of the selected feedstock production projects, and Fig. 1, their location. The different types of LUCC in each site are outlined in a study that assessed carbon stock changes following the land conversion to feedstock production^8^.

The project site in Dwangwa (Malawi) contains a large-scale sugarcane plantation and a sugar mill operated by a multinational company (Illovo) since the late 1970s. The core estate is surrounded by irrigated and rainfed sugarcane plots managed by smallholders that sell sugarcane to the Illovo mill. The irrigated smallholders are part of the Dwangwa Cane Growers Limited (DCGL). In contrast the rainfed smallholders are part of a growing number of smallholder associations^21^. A fully Malawi-owned company, EthCo Malawi, operates a distillery that uses molasses purchased from the Illovo mill for ethanol production.

The project site in Tshaneni (Swaziland) contains a large sugarcane estate and two mills (in Mhulme and Shimunye) operated by the Royal Swazi Sugar Company (RSSC). RSSC also operates an ethanol distillery that uses the molasses from the two mills for ethanol production. Sugarcane production started in 1958 and has expanded considerably in the past decades. A smallholder community development programme was promoted by the Swaziland government (SWADE) in the late 1990s to develop capacity for irrigated sugarcane production among smallholders near the two mills^19^. Smallholders were incentivised to pool their cropland for sugarcane production and formed 28 associations. These associations operate as independent cooperatives or companies. The involved smallholders are equal partners in the associations and receive annual dividends by selling sugarcane to the RSSC mills^19^.

The project site in Mangochi (Malawi) contains a few hundred smallholders that produce jatropha in hedges along the boundaries of their small family farms. A private company, BioEnergy Resources Ltd (BERL), promoted this model of jatropha production assuming that farm boundaries are underutilized^9,22^ Planting jatropha in these hedges was expected to have minimal effects on food crop production. Though BERL targeted around 1,00,000 farmers across Malawi^23^, only a fraction of farmers took up and maintained jatropha production in this way. BERL introduced jatropha in Mangochi in 2007, making it one of the first areas of jatropha production in the country. By the time of data collection jatropha farmers in the area had already sold seeds to BERL over 3 production cycles.

The site in Buzi (Mozambique) contains a large-scale jatropha plantation operated by a private company (Niqel) established in 2006–2007. The initial plan was to convert 10,000 ha of miombo woodland for jatropha production, but at the time of data collection, only about 1,700 ha had been planted (Table 1). Niqel aimed to process jatropha oil to fuel, and jatropha seedcake into fertiliser, animal feed and organic pellets. In this study area only the large Niqel plantation produced jatropha, with no smallholders being linked to the project.

Maputo is the only major city in Africa where ethanol for cooking moved beyond small, pilot projects. Cleanstar, a private company, promoted extensively ethanol stoves and ethanol fuel for cooking in Maputo city. This was to capitalise on the pre-existing efforts of another company called NDzilo. Cleanstar initially received extensive interest and large amounts of capital from international donors, but collapsed in 2013. Before its collapse it had managed to sell approximately 30,000 ethanol stoves in the wider Maputo city area^16^, which consumed an estimated 70,000–1,40,000 L of ethanol per month. Cleanstar also initiated ethanol production in a state-of-art distillery in the city of Beira (Central Mozambique) using cassava sourced from smallholders in the northern Mozambique. A large fraction of these ethanol stoves were still operational (i.e. using ethanol imported from South Africa) at the time of the survey, despite Cleanstar’s demise.

### Household Survey

We undertook a structured household survey to collect data to assess the local impacts of biofuel feedstock production on livelihoods, ecosystem services, food security and poverty alleviation at the study sites. Households with different types of involvement in feedstock production were targeted, such as plantation workers and feedstock smallholders (intervention groups) and households not involved in feedstock production (control groups) (see next section for sampling procedures).

The household survey consisted of 15 sections. Nine sections were relevant to all respondent groups and the remaining 6 only to some groups as identified below:

- Household demographic and socioeconomic profiles (All respondent groups)
- Income and livelihoods (All respondent groups)
- Multi-dimensional poverty (All respondent groups)
- Energy access and use (All respondent groups)
- Perceived provision of ecosystem services (All respondent groups)
- Agro-economic practices (All respondent groups)
- Food security (All respondent groups)
- Experience/impacts of sugarcane farming (Only for relevant intervention groups in Dwanwa and Tshaneni, see Table 1)
- Employment benefits of sugarcane and jatropha (Only for relevant intervention groups in Dwangwa, Tshaneni and Buzi, see Table 1)
- Communal impacts of sugarcane plantations (Only for relevant control groups in Dwangwa and Tshaneni, see Table 1)
- Experience/impacts of jatropha farming (Only for relevant intervention groups in Mangochi, see Table 1)
- Perceptions of jatropha growing (Only for control group in Mangochi, see Table 1)
- Communal impacts of jatropha plantations (Only for relevant control groups in Buzi, see Table 1)
- Subjective wellbeing (All respondent groups)
- General comments (All respondent groups)

The household survey included both close-ended and open-ended questions. In particular, it contained generic questions about household demographics, livelihood options, agro-economic practices, reliance on ecosystem services and energy access/use patterns. The survey also contained questions to calculate the multi-dimensional poverty index (MPI), which is a composite measure of poverty^24^. The overwhelming majority of the questions were not related to perceptions. We also ensured that the few perception questions that did exist (mainly related to ecosystem services) were framed in a general manner to avoid gender-differentiated responses. In particular, such questions elicited the importance of ecosystem services for households rather than individual respondents.

Questions from Sections A-G, N and O were asked of all groups (see next Section). Questions in these sections were mainly closed-ended and employed fixed ranges that were coded appropriately. Questions in Sections H-M varied between groups, and focused on the differentiated experiences/impacts of involvement (or non-involvement) in feedstock production. Most of the open-ended questions of the household survey belonged to these sections (and also part of Section O). Apart from the questions and the answer options, the questionnaires provided enumerators with clear instructions on how to capture each answer.

We also undertook a structured household survey to identify the factors that influence the adoption and sustained use of ethanol stoves in Maputo city. The vast majority of the questions in this household survey were closed-ended using fixed ranges that were coded appropriately. We targeted areas of Maputo city that experienced a substantial promotion and adoption of ethanol stoves. Our sample consisted of households that used ethanol and households that used charcoal as the main cooking option (see Section below). The household survey contained 5 sections as identified below:

- Household demographic and socioeconomic profiles (All respondent groups)
- Energy use patterns (All respondent groups)
- Ethanol stove use patterns (Only for ethanol stove users)
- Charcoal stove use patterns (Only for charcoal stove users)
- Poverty indicators and income (All respondent groups)

### Sampling strategy

We surveyed households with differentiated levels of involvement in feedstock production. This included groups that were involved in feedstock production (i.e. households of plantation workers or feedstock smallholders) and control groups not involved in feedstock production (i.e. households that did not contain plantation workers or feedstock smallholders).

We sampled different intervention and control groups in each study site, in order to capture the unique characteristic of each area and feedstock production mode (Table 2). Particularly, in the study sites that contain large plantations (i.e. Dwangwa-Malawi, Buzi-Mozambique, Tshaneni-Swaziland), we sampled two control groups: (a) a control group in the vicinity of the plantation, and (b) a control group further away (see below). We used this sampling approach to collect data that could be used to establish whether living in the proximity of plantations had any positive or negative spillover effects, including benefits from infrastructure (e.g. roads, clinics, secondary job creation) or disadvantages from the loss of agricultural land and natural areas^25^. In total, we sampled 1,544 households across all feedstock production sites (Table 2). We provide details on the sampling strategy followed at each study site below.

In Dwangwa (Malawi), we sampled three intervention groups and two control groups (Table 2). The intervention groups consisted of: (a) formal plantation workers (working for Illovo); (b) irrigated sugarcane smallholders, and (c) rainfed sugarcane smallholders. We also sampled two control groups, one consisting of subsistence farmers living in the vicinity of the sugarcane-growing areas, and the other consisting of subsistence farmers living approximately 50 km from the sugarcane belt. As sugarcane is a highly perishable crop, this distance is often considered the point after which it becomes uneconomical to supply sugarcane to a mill^26^.

We sampled permanent plantation workers using a list of employees provided by the human resources office of Illovo. We randomly selected workers below mid-level management for in-person interviews. Similarly, we obtained lists of irrigated and rainfed smallholders from the various associations of irrigated and rainfed farmers operating in the Dwangwa area. To avoid oversampling respondents from any specific association, we randomly selected respondents based on the overall number of farmers belonging to each farmer association, using weights proportionate to the sample size. In total, we surveyed 104 irrigated sugarcane smallholders from the following four associations (see also variable “H6_specify”): Bua (N=2), DCGL-A (N=71), Tipate (N=8) and Umbdzi (N=23). We also surveyed 107 rainfed sugarcane smallholders from the following seven associations (see also variable “H6_specify”): Bua (N=6), DCGL-A (N=25), Tipate (N=18), Umbdzi (N=14), Green Leaf (N= 6), Independent (N=8), and Kabadwa (N=30).

In Tshaneni (Swaziland), we sampled three intervention groups and two control groups (Table 2). Intervention groups included: (a) formal workers of the large-scale RSSC plantation, (b) workers of the irrigated community plantations, and (c) irrigated sugarcane smallholders. Plantation workers were sampled using the full list of employees obtained from the office of human resources of RSSC. We randomly selected respondents below mid-level management for in-person interviews. Sugarcane smallholders were selected in a two-stage process. First we randomly selected 14 associations from the 27 smallholder associations operating in the area. Then we visited each of the 14 associations and obtained a list of all smallholders belonging to the association. In order to avoid oversampling respondents from any specific association, we then randomly selected a set of respondents based on the overall number of farmers belonging to each farmer association using weights proportionate to sample size This two-stage sampling process was adopted to allow for a good spread within the sugarcane production landscape, without diluting sample sizes by surveying all of the 27 associations.

In total, we surveyed 93 irrigated sugarcane smallholders from the following 14 associations (see also variable “H6_specify”): Buhle Besive (N=4), Intamakuphila (N=21), Mabhudvu (N=7), Mangweni (N=11), Phakama (N=16), Sivukile (N=4), Vuka (N=12), Vukutimele (N=21), Vuka Siduwashini (N=4), Makhabeni (N=6), Manknjane (N=2), Phakama Mafucula (N=3), MMN (N=2), and Ntisheni (N=1).

In Mangochi (Malawi), we sampled one intervention group (i.e. jatropha smallholders) and one control group (Table 2). We did not sample a faraway control group as there was no large-scale jatropha plantation in the study site that could have had positive or negative spill-over effects. We selected respondents that still sold jatropha seeds based on information provided by lead farmers. Lead farmers in the Mangochi area were identified through information provided by BERL. We completed surveys only with those households that had already sold jatropha to BERL between 2 and 3 times.

In Buzi (Mozambique), we sampled one intervention group (i.e. formal Niqel workers) and two control groups (Table 2). Due to the smaller size of the Niqel jatropha plantation in terms of area and workforce (compared to the Illovo and RSSC sugarcane plantations), workers were randomly selected from sites where people congregated such as the garage, nurseries and picking points. Interviews were conducted in the working areas during breaks, or before/after shifts. Control groups adjacent to the jatropha plantation were randomly selected through transect walks that started from randomly selected points around the plantation. Faraway control sites were identified following the procedure described in the previous section. Respondents in these faraway control sites were, wherever possible, identified randomly through transect walks. We interviewed every second household encountered along the transect walks to allow for some level of randomization considering the very low population density and the high forest density^8,9^. Most respondents of the control households were located close to the road network. In some areas, clusters emerged due to the relatively higher population densities. These included clusters C1-C8 for the close control (Group 13) and F1-F4 for the faraway control (Group 14), see variable “Other_comments”.

The survey in Maputo city targeted 341 households from the neighborhoods of Benfica (N=72), Chamaculo (N=61), Hulene (N=60), Mavalane (N=69), Maxaquene (N=65), and Urbanizacao (N=14). Of these, 58 households (17%) had adopted and sustained ethanol stove use, 42 households (12%) had adopted but discontinued ethanol stoves, and 241 households (71%) never adopted ethanol stoves. We defined adopters as those households that, at one point in the past, obtained and used an ethanol stove. Sustained users were classified as those households that had adopted an ethanol stove and continued to use it as the primary or the secondary cooking option up to the time of the survey^27^. Quitters were classified as those households that have/had an ethanol stove but stopped using it^27^. Non-adopters were classified as those households that never possessed an ethanol stove^27^.

Our household survey was carried out in specific study areas within Maputo, which had experienced an extensive adoption of ethanol stoves. This was indicated through expert interviews with the management of the NDziLO Company that spearheaded the stove promotion campaigns. We did not aim to provide a baseline of cooking fuel preferences across the city through this purposeful sampling, but rather aimed to capture the factors that influenced the adoption and sustained use of ethanol stoves (e.g. cost, use characteristics) when considering the other available cooking options (e.g. charcoal) through the perceptions of respondents. The household survey focused mainly on user perceptions to elicit the factors that influence stove adoption and use. The survey also contained key demographic and socioeconomic data to study adoption dynamics. Stove adopters in these areas were randomly sampled from the sales records of NDziLO, and the neighbours of adopters were selected as the non-adopters (see below).

The selected respondent for the household survey in the feedstock production sites was one of the key decision-makers within each household. The respondent was essentially either the household head or the spouse, whoever of the two was available at the time of the visit. By targeting the main decision-makers in the household, we aimed to avoid gender imbalance in the sample. We undertook interviews only with one respondent per household, as in the rural contexts of Africa male respondents tend to dominate the discussion if both genders are present. Pre-testing of the questionnaire enabled the research team to verify whether the questions and formulations were understandable to both male and female household decision-makers (see below).

For the household survey in Maputo city, we targeted the main female decision-maker within the household, as this was usually the household member mostly involved in food preparation and fuel/stove procurement. On those occasions that the main female decision-maker was not available, we sought information from the spouse or another person that was extensively involved in the daily cooking.

## Data Records

The data records and other relevant documents are available in English through the UK Data Service repository under safeguard access (Data Citation 1). The data records were collected during the research project *“Unraveling biofuel impacts on ecosystem services, human wellbeing and poverty alleviation in Sub-Saharan Africa*”. All material associated with these datasets can be downloaded (Data Citation 1) as the excel files:

- ESPA_Biofuels_Dataset_production.xlsx: contains the responses of the household surveys at the four feedstock production sites (i.e. Dwanwa, Tshaneni, Mangochi, Buzi);
- ESPA_Biofuels_Dataset_adoption.xlsx: contains the responses of the household survey at Maputo city.

The archived material also contains the actual household surveys and the associated information provided to enumerators and interviewed households. The material exists as:

- ESPA_Biofuels_Questionnaire_production.pdf: contains the household survey for the four feedstock production sites using a description for each variable name and the question ID;
- ESPA_Biofuels_Questionnaire_adoption.pdf: contains the household survey for Maputo city using a description for each variable name and the question ID;
- ESPA_Biofuels_Consent_form.docx: contains the information offered to the targeted households when seeking oral consent to proceed with the interview.

## Technical Validation

It is important to reduce sampling and non-sampling errors in large-scale household surveys related to agriculture^28^. To ensure high data quality, we implemented several measures to avoid biases in the selection of the surveyed households, as well as, errors in data collection and transcription as discussed below.

### Reducing sampling errors

We developed a comprehensive sampling strategy to ensure reliable and unbiased identification of responding households. This was done for both the intervention and control households (see previous section).

To identify those households involved in feedstock production (i.e. intervention groups), we relied extensively on employee and farmer lists obtained after negotiations with the different companies (i.e. BERL, RSSC, Illovo), lead farmers, and farmer associations. To ensure a relatively large and balanced pool of respondents, we randomly selected approximately 120 respondents from each intervention category through these lists. Of these, we initially selected 100 respondents to undertake the survey, with the rest acting as substitutes to replace households that either declined to be involved in the survey or were unable to provide the required information within the time frame of the survey. We ensured the effective randomisation of the sample and avoided biases that could have emerged through non-randomised techniques, by relying on such comprehensive lists to draw random samples that met the study criteria.

The sugarcane companies and outgrower schemes in the study areas tend to be well organized and managed^19,21^, so the coverage of the lists received for the workers (i.e. from Illovo, RSSC) and the sugarcane smallholders in Dwangwa and Tshaneni is complete and comprehensive. We also used the latest available information found in lists from the farming season before the survey to avoid coverage errors. In comparison, the lists of jatropha smallholders in Mangochi (Malawi) were less comprehensive and subject to change, as several farmers that initially adopted jatropha, discontinued its production due to various circumstances. In order to locate those farmers that had not ceased jatropha production, we cross-checked lists with information provided by lead farmers and other interviewed jatropha smallholders. For plantation workers in Buzi (Mozambique), we sampled randomly workers from the main points of congregation such as the garage, nursery and picking points (rather than relying on worker lists) due to the much smaller workforce and concentrated area of the operations compared to sugarcane plantations in Dwangwa and Tshaneni. In total, we surveyed about a third of the permanent Niqel workers.

The nearby control groups were selected from the neighbours of those involved in feedstock production in Dwangwa (Malawi), Tshaneni (Swaziland) and Mangochi (Malawi) (i.e. Table 1). This approach facilitated a good spread of control groups in the feedstock production areas and similarly provided a randomised comparative sample. The only exception was Buzi (Mozambique), where, we relied on transect walks to identify the control group around the plantation due to the low population density and high forest density, (see “Sampling Strategy”).

We followed a two-step procedure to identify the control groups far away from the feedstock production areas (i.e. far away control, Table 1). First we identified the control sites and then the respondent households. Faraway control sites were identified based on ability to grow the feedstock (i.e. agro-ecological similarity), and similarity in landscape characteristics and livelihood activities. In particular, the control site in Dwangwa (Malawi) was situated an area that was earmarked for sugarcane expansion, with plans to commence sugarcane production in 2016. Control sites in Tshaneni (Swaziland) and Buzi (Mozambique) were selected based on satellite images, local interviews and site visits by team members and local partners to verify the similarity criteria with the intervention areas. Considering that the main non-feedstock-related livelihoods strategies in the intervention areas were related to subsistence agriculture, far away control sites and control group respondents were also subsistence farmers to ensure a proper comparative basis. For this reason, we avoided relatively urbanised areas or market posts where respondents might have relied on other livelihood activities. Once the control site was selected, we randomised the selection of respondent households through transect walks (see “Sampling Strategy).

An important methodological decision was to avoid sampling households that had two types of involvement in feedstock production value chains. This included, for example, households in Dwangwa (Malawi) and Tshaneni (Swaziland) whose members were both permanent plantation workers and sugarcane smallholders. This was achieved by asking straightforward questions on the household’s involvement in sugarcane value chains at the beginning of the interview. If a dual-status was observed, the enumerators were instructed to stop the interview and proceed to the next household.

For the household survey in Maputo city, respondents were selected from client lists provided by NDziLO that spearheaded the ethanol stove promotion campaigns. We randomly selected ethanol stove buyers in the different study areas described in the previous section. Due to the type of information received from NDziLO (i.e. lists of stove purchases rather than ethanol fuel purchases), we did not know in advance the households that had stopped using the ethanol stoves. Thus, while we were able to sample in advance stove adopters, we were unable to identify stove quitters (see “Sampling Strategy”). However, given the randomisation process followed in the household selection, we believe that this approach has not inserted biases in the sampling process.

The interviews for all surveys were conducted in-person and in the local language, and in most cases at the respondents’ residence. This was done to achieve a high response rate and undertake the interview in settings that were familiar and comfortable for respondents, as some questions could be deemed sensitive (e.g. income, perceptions of employment, household mortality rates). The only exceptions were interviews with workers in Buzi (Niqel), Dwangwa (Illovo), and Tshaneni (RSSC, community plantations). Some interviews were performed on company grounds during breaks or before/after shifts. There were also a couple of interviews with faraway control group respondents in Buzi that were done at the local chief’s house. We identify in the last column of the dataset, which interviews were not conducted at the respondents’ residence (see variable “Other_comments”). The numbers of interviews for each group are as follows: are:

- Group 1 (Illovo plantation workers in Dwangwa, Malawi): 12 out of 104 interviews
- Group 8 (RSSC plantation workers in Tshaneni, Swaziland): 75 out of 103 interviews
- Group 15 (community plantation workers in Tshaneni, Swaziland): 108 out of 113 interviews
- Group 14 (far away control group in Buzi, Mozambique): 2 out of 108 respondents

We did not record statistics related to response rates. However, we achieved very high response rates for all study groups both in the feedstock production sites (i.e. Dwangwa, Tshaneni, Mangochi, Buzi) and Maputo city. The main reason for declining to participate in the survey was the inability to offer time as data collection in some sites was conducted around important periods of the year such as land preparation and cultivation in anticipation of the rainy season (e.g. in Buzi, Mozambique). If, for some reason, the survey was requiring more time than anticipated (and the respondents requested to halt the survey), we received permission to visit the household the following day to resume and finalise it. If that was not possible, we discarded the survey. However this was done in a very small minority of the cases.

### Reducing non-sampling errors

The most important types of non-sampling errors are non-response errors and measurement errors. These can occur if interviewers deviate from the established study design, influence the respondents, or the respondents fail to remember/report details properly^28^.

To reduce non-sampling errors, we developed a comprehensive protocol to design and implement at the household surveys. As explained in more detail below, we took measures related to the:

- design of the household survey;
- translation, pre-testing and revision of the questionnaire;
- selection and training of enumerators;
- definition of households and selection of respondents;
- triangulation of impact mechanisms and relevant findings.

The research team members responsible for the design of the household survey (i.e. the first six authors of this paper) visited all four sites prior to the development of the survey. Between them, the research team members had prior experience conducting research in three out of the four study sites^9,11^. The actual development of the draft household survey was undertaken during preliminary visits to the three study sites in Swaziland and Malawi. We held expert interviews and performed in situ observation during these site visits to understand the unique site characteristics, as well as the dynamics between local communities and feedstock production. For the development of the household survey, we followed an iterative process where site visits and survey development alternated in order to integrate each new insight. At the end of each site visit, every draft questionnaire was piloted in two to three local households, with members of the team present to appreciate the survey length and the type of obtained responses.

All these insights were considered when developing the final survey in the 1.5 months following the preliminary site visits (“see Household Survey”). The survey also contained italicised guidelines on how to administer each question, and which questions to check for internal consistency. The household survey was translated to the main local language in Malawi (Chichewa) and Swaziland (SiSwati). The Malawi translation was performed by research team members, while in Swaziland it was conducted by hired consultants that have had extensive experience working in the sugarcane projects in Tshaneni and Dwangwa. Due to the lack of a written form for the local language in Mozambique, the research team translated the questionnaire to Portuguese. The final translated version in the local languages was agreed and finalised during the training of the enumerators and pre-testing of the questionnaire for all sites (see below).

Selection of enumerators in each site was based on their ability to speak the local language (and local dialect were needed), be conversant in English (or Portuguese in Mozambique), and where possible, previous experience conducting household surveys. This was to ensure the effective communication between the enumeration and the research teams. At each site, enumerators were trained by at least one research team member that designed the questionnaire (see above). Initially, the research team member(s) went through the survey with the selected enumerators to thoroughly explain the design of the survey, its individual components, and the overall aim of the fieldwork. This initial training was used to fine-tune the format of some questions and the local translations (see below). The activity was undertaken off-site to allow for the maximum concentration of the research and the enumeration teams.

Subsequently, the household survey was pre-tested (approximately 15 surveys per site) to identify site-specific dynamics, the ability of participants to answer all questions, and the local terminology, as well as to train the local enumerators under real conditions. We identified that both male and female respondents were comfortable answering all questions during the pre-testing.

Based on this pre-testing, we changed the formulations slightly (as well as the translation of some questions) to reflect the unique characteristics of each site. These changes were made after reaching consensus within the research and the enumeration teams, and were kept to a minimum to allow for the greatest possible comparability between sites. During this two-stage training process, we performed the final selection of enumerators based on their proven ability to administer the questionnaire, as well as their commitment to the process.

To ensure the quality of the data collection processes and the effective supervision of enumerators, research team members were present during the entire data collection campaign at each site. This quality control included checking each collected questionnaire and re-training enumerators if errors were found. Senior team members also frequently attended the data collection activities in the field, to ensure the sampling and data collection protocol was being followed. Enumerators were asked to perform internal consistency checks on-the-spot through checking combinations of certain questions, and asked again specific questions if inconsistencies were found. Questionnaires that did not meet data quality requirements were discarded at the end of each day following cross-checking by a research team member.

Prior to digitisation, research team members checked again the completed questionnaires for completeness. Questionnaires were then digitised using a customised Microsoft Access database developed by the research team to ensure data entry was performed smoothly and error-free in a user-friendly environment. Fields in the Access database were limited to appropriate values to reduce data entry errors. Research team members thoroughly checked all entries and identified outliers and cleaned the data, where needed, by consulting the original paper surveys. The original paper versions of the household survey have been stored following the requirements of UK Economic and Social Science Research Council (ESRC) as a means of allowing the research team to check individual entries if, and when, necessary.

Finally, given the time/budget constraints and large sample sizes, multiple site visits were not performed in feedstock production areas, as a means of further reducing memory and recall biases. To cross-check the mechanisms of change and some of the obtained data, we also ran several focus group discussions (FGDs) in each study site. These FGDs aimed to elicit community perceptions on some of the studied themes. The actual number of FGDs and involved participants varied between feedstock production study sites, but included local community members that were both involved and not involved in feedstock production activities. The FGDs elicited community perceptions (i.e. they were not framed at the household level, but at the community level), and focused on the broader landscape effects of feedstock production mainly related to land use change, ecosystem services and human wellbeing. Such combinations of FGDs with household surveys is very common in comparable studies in African contexts^29,30^. In most cases, we undertook different FGDs with male and female respondents, as some of the landscape-level impacts of feedstock production can be gender-differentiated^31^. In all cases, the findings of the FGDs supported the broader impact mechanisms considered during the development of the survey, as well as some of the relevant findings of the household surveys.

### Ethical approval

The research protocol was designed following the good practice recommendations from the UK Economic and Social Science Research Council (ESRC). The research protocol was reviewed and approved by the University of Oxford Central University Research Ethics Committee (CUREC) that oversees the university’s ethical review processes. This procedure was finalised and granted approval in June 2014, before the full surveys were undertaken.

Prior to contacting local community members to participate in the study, we undertook extensive meetings with traditional authorities, and representatives of the private companies and farmers’ associations. This was important to gain their support for the study and entry into the local communities.

All selected respondents were interviewed after orally providing informed consent on the information in the consent forms approved by CUREC. We ensured that all respondents were fully informed about the aims of the research and thoroughly understood what their participation in this study entailed. We sought oral consent, as large segments of the local communities in the study sites were illiterate. Participation was voluntary with respondents reserving the right to decline to be interviewed. All potential respondents were informed that refusal to participate in the household survey would not lead to any adverse consequences. No payments were made to respondents.

Finally, surveys were anonymised to avoid the identification or discrimination of respondents. For this reason, we have withheld GPS coordinates and omitted open text comments that could be used to identify the respondent or disclose personal/sensitive information. These comments were changed with the phrase “*answer withheld to protect anonymity and/or personal information*” (see variable “Res_comments” and “Enu_comments”).

## Usage Notes

The datasets (Data Citation 1) are relevant for studies that explore the local impacts of different bioenergy options and/or industrial crops in Africa. The dataset from the feedstock production sites (i.e. Dwangwa, Tshaneni, Mangochi, Buzi) is particularly relevant for studies that will seek to compare the impacts of different biofuel feedstocks, modes of production, or ways of organizing feedstock/biofuel production chains. Beyond biofuels/bioenergy, this dataset is relevant to studies that explore the impacts of different agricultural practices in Africa, especially those related to cash crops and industrial crops.

However, some caution needs to be applied when utilizing this dataset due to issues of comparability between groups and sites, and possible errors due to respondent recollection. Whilst we have used consistent versions and translations of the same survey between sites to reduce non-sampling errors (see above), we used different sampling techniques for the intervention and control groups within sites. This was necessary, as the research team did not have consistent information for the different groups between study sites. We believe that all sampling methods are robust, however, and can allow for a high degree of randomization. Additionally, the dataset is based on the respondents’ recollections, and is therefore subject to the normal constraints that such elicitation methods entail. Even though the absolute values may be uncertain, to some extent, we believe that the comparison of patterns between groups can allow for the identification of group-based trends within the same study site. Interested users are diverted to other publications that have used this dataset^10,32,33^.

We recommend using this dataset for explaining patterns between groups and the impact mechanisms within the same site, rather than for undertaking direct comparisons between sites. Despite our efforts to use the same protocol at all sites, comparison between sites should be done with caution due to the (a) spread of projects between countries, (b) sampling discrepancies, and (c) timing of the fieldwork.

Regarding (a), while the selected feedstock production sites reflect well the different types of biofuel projects in Africa^1,5,6^, it was not possible to locate all the different biofuel project configurations within the same country. This was due to the different biofuel policy priorities among countries in southern Africa and the collapse of the jatropha sector^1,17^. To some extent, these can reduce the comparability of results between countries, as the projects operate in different environmental and socioeconomic contexts. The different national and local circumstances should also be taken into consideration when attempting comparisons between sites. Furthermore, the spread of sites across different countries made it impossible to link the studies from the production to the demand-side. This precludes a more comprehensive view of biofuel value chains in southern Africa.

Regarding (b), it was not always possible to follow the same sampling protocol among groups with the same involvement in feedstock production (e.g. plantation workers, smallholders, control groups). Such sampling method discrepancies were due to the different operational characteristics of the biofuel projects, the unique socioeconomic/environmental context of each locality (e.g. population density), and the information available to the research team. We believe, however, that the sampling protocols are individually robust and that they can allow for a good level of randomization for each group.

Regarding (c), the timing of the fieldwork can affect the levels for some variables that have a fixed recollection period or that are sensitive to the period of the year. For example, the surveys in Malawi were conducted during periods of relatively good food availability close to harvest (August-September 2014), but the surveys in Mozambique (November-December 2014) and Swaziland (March-April 2015) were conducted during periods of relatively lower food security.

The dataset from Maputo can be relevant to studies that explore the factors influencing the adoption and use of household cooking options in developing contexts, Particular application fields include clean cooking options in urban contexts, African contexts, or both. It is worth noting that due to its focus on perceptions, this dataset might have a lower usability in studies that aim to elicit broader socioeconomic drivers of stove/fuel adoption. However, the dataset includes key household demographic and socioeconomic variables that can be used to study to some extent socioeconomic drivers of stove/fuel adoption.

## Additional information

**How to cite this article**: Gasparatos, A. *et al*. Survey of local impacts of biofuel crop production and adoption of ethanol stoves in southern Africa. *Sci. Data* 5:180186 doi: 10.1038/sdata.2018.186 (2018).

**Publisher’s note**: Springer Nature remains neutral with regard to jurisdictional claims in published maps and institutional affiliations.

## Supplementary Material



## Figures and Tables

**Table 1:** Description of study sites and biofuel projects.

**Site**	**Feedstock**	**Type**	**Year started**	**Data collection**	**Project extent**	**Soil type**	**Harvest**	**Other details**
**Dwangwa, Malawi**	Sugarcane	Hybrid. Core large plantation (Illovo), surrounded by irrigated and rainfed sugarcane smallholders	Core plantation: 1977Irrigated smallholders: early 1990sRainfed smallholders: unknown	Aug-Sep 2014	Core plantation and irrigated smallholders: 110 km^2^Rainfed smallholders: unknown	Sandy and clay loam	May-June	Molasses from Illovo mills are sold to EthCo as a feedstock for ethanol production
**Tshaneni, Swaziland**	Sugarcane	Hybrid.Core large plantation (RSSC) surrounded by irrigated sugarcane community plantations.	Core plantation (Mhlume): 1958Core plantation (Shimunye): 1979Irrigated community plantations: 1999	Nov-Dec 2014	Core plantations: 220 km^2^Community plantations: 55 km^2^ involving 27 associations with 2387 farmers	Core plantations: Red brown clay loamCommunity plantations: Sandy and clay loam	May-Jun	Molasses from the two RSSC mills are used as feedstock for ethanol production. SWADE, a parastatal agency, assisted the development of smallholder irrigated sugarcane production for rural development
**Mangochi, Malawi**	Jatropha	Smallholder	2008	Oct 2014	About 800 farmers in study area	Loamy sands or sandy loams	Dec-May	Farmers sell jatropha seeds to BERL for processing into biofuel. Jatropha oil was produced by BERL for a small period of time,
**Buzi, Mozambique**	Jatropha	Large plantation (Niqel)	2008	Mar-Apr 2015	17 km^2^	Sands at top, heavy clays at the bottom	Feb-Apr	First jatropha seeds harvested in 2012. Plans to produce jatropha oil in 2015 but unclear whether that eventually happened.

**Table 2:** Number of household surveys for each study group and site.

	**Dwangwa, Malawi (sugarcane)**	**Tshaneni, Swaziland (sugarcane)**	**Mangochi, Malawi (jatropha)**	**Buzi, Mozambique (jatropha)**
Biofuel crop farmers (irrigated)	104 (Group 2)	93 (Group 9)	NA	NA
Biofuel crop farmers (rainfed)	107 (Group 3)	NA	101 (Group 6)	NA
Workers in large plantations	104 (Group 1)	103 (Group 8)	NA	98 (Group 12)
Workers in community plantations	NA	113 (Group 15)	NA	NA
Not involved, nearby control group	104 (Group 4)	101 (Group 10)	101 (Group 7)	104 (Group 13)
Not involved, far away control group	99 (Group 5)	104 (Group 11)	NA	108 (Group 14)
**TOTAL**	***518***	***514***	***202***	***310***
Note: Group numbers correspond to variable “Respondent_Type”. NA denotes that due to the unique project characteristics this group was not present in the particular site.				
